# Comparison of the Effect of Adding Dexmedetomidine Versus Dexamethasone to Bupivacaine in Transverse Abdominis Plane Block on Postoperative Pain Intensity in Patients Undergoing Laparoscopic Cholecystectomy

**DOI:** 10.5812/aapm-162462

**Published:** 2025-07-15

**Authors:** Alieh Zamani Kiasari, Ramin Razavi, Samira Sobhani, Negar Shirvani Ghadikolaee, Nasimalsadat Mousavi Khorshidi, Keihan Shabankhani, Nafiseh Faghani-Makrani

**Affiliations:** 1 Department of Anesthesiology, Faculty of Medicine, Mazandaran University of Medical Sciences, Sari, Iran; 2 Department of Anesthesiology, Faculty of Medicine, Golestan University of Medical Sciences, Gorgan, Iran

**Keywords:** Bupivacaine, Dexamethasone, Dexmedetomidine, Transverse Abdominis Plane Block

## Abstract

**Background:**

Laparoscopic cholecystectomy, compared to open surgery, offers advantages such as lower pain levels and shorter hospitalization. However, postoperative pain remains a common challenge. Inadequate pain control may lead to discomfort, reduced mobility, and prolonged hospitalization. This study aimed to compare the effect of adding dexmedetomidine versus dexamethasone to bupivacaine in transverse abdominis plane (TAP) block on postoperative pain intensity in patients undergoing laparoscopic cholecystectomy.

**Objectives:**

The present study aimed to compare the efficacy of dexmedetomidine and dexamethasone as adjuvants to bupivacaine in ultrasound-guided TAP blocks for laparoscopic cholecystectomy. Primary outcomes included sensory block duration, postoperative pain scores, 24-hour morphine consumption, and time to rescue analgesia. Secondary outcomes included sedation levels, hemodynamic stability, and incidence of adverse events.

**Methods:**

This randomized, double-blind, controlled clinical trial included 120 ASA I-II patients aged 18 - 65 years, allocated into three groups: Bupivacaine alone, dexamethasone + bupivacaine, and dexmedetomidine + bupivacaine. Pain intensity, morphine consumption, time to first analgesia, block characteristics, hospital stay, and complications were evaluated.

**Results:**

The addition of dexmedetomidine or dexamethasone to bupivacaine in ultrasound-guided TAP blocks significantly improved postoperative outcomes. Compared to the control group (bupivacaine alone), both adjuvants reduced pain intensity (VAS scores, P < 0.001) and 24-hour morphine consumption (P < 0.001), with dexmedetomidine demonstrating superior efficacy. Sensory block duration was prolonged in the dexmedetomidine (330 minutes) and dexamethasone (180 minutes) groups versus control (155 minutes; P < 0.001). Hospital stays were shortest in the dexmedetomidine group (1 day vs. 2.5 days control; P < 0.001).

**Conclusions:**

Adding dexmedetomidine or dexamethasone to bupivacaine in TAP block enhances analgesia and shortens hospital stay following laparoscopic cholecystectomy.

## 1. Background

Laparoscopic cholecystectomy is one of the most common minimally invasive surgical procedures performed for symptomatic gallstone disease (1). Compared to open surgery, laparoscopic cholecystectomy offers clear advantages; however, postoperative pain remains a frequent complaint (2, 3). Patients undergoing laparoscopic cholecystectomy experience significant postoperative pain. In addition to causing patient discomfort, extended immobilization, thromboembolism, and pulmonary complications, chronic pain may lengthen hospital stays if left untreated (4). Post-laparoscopic cholecystectomy pain is primarily caused by trocar insertion sites, abdominal wall stretching due to pneumoperitoneum, and hepatic bed disturbances caused by cholecystectomy (5). This pain can be managed through various methods, including the use of non-steroidal anti-inflammatory drugs (NSAIDs) such as ketorolac (6), preventive analgesic regimens containing ketamine, intraperitoneal local anesthetics, local incision infiltration, regional anesthesia techniques such as the transverse abdominis plane (TAP) block (7), patient-controlled thoracic epidural analgesia, patient-controlled intravenous analgesia, opioids, and multimodal analgesia (8, 9).

A peripheral nerve block called the TAP block is used to anesthetize the nerves that supply the anterior abdominal wall (T6 to L1) (10). This block was first described by Rafi in 2001 (10). It can be performed by injecting local anesthetics into the fascial plane between the internal oblique and transversus abdominis muscles using a blind technique based on superficial anatomical landmarks (11, 12) or, more recently, under ultrasound guidance with direct visualization (13, 14). The TAP block is currently a safe and efficient method for lowering postoperative pain in abdominal surgery due to the growing use of ultrasound guidance for accurate needle localization (15). The duration of the TAP block is limited by the effect of local anesthetics. Recently, adjuvants have been added to local anesthetics, as previously described, to prolong the effect of the TAP block (16).

Bupivacaine, ropivacaine, and levobupivacaine are local anesthetics commonly preferred for TAP blocks (17). A wide range of compounds, such as opioids, benzodiazepines, α2-adrenergic agonists, N-methyl-D-aspartate (NMDA) receptor antagonists, dexamethasone, neostigmine, and magnesium sulfate, have been used as adjuvants to local anesthetics in peripheral nerve blocks to increase analgesic effects and extend block duration (18). Bupivacaine is a local anesthetic capable of providing high-quality, long-lasting postoperative analgesia (19). The TAP block using bupivacaine significantly reduces pain scores (20).

The use of dexmedetomidine with bupivacaine in peripheral nerve blocks is associated with prolonged local anesthetic effects. Because of its analgesic, sedative, anesthetic-sparing, sympatholytic, and hemodynamic-stabilizing qualities, α2-adrenergic receptors have been used as adjuvants to local anesthetics to prolong their duration. Dexmedetomidine, an imidazole derivative and stereoisomer of medetomidine, is a highly selective α2-adrenergic receptor agonist with a high α2/α1 activity ratio (1620:1 compared to 220:1 for clonidine) (21). This agent provides dose-dependent analgesia and anxiolysis without respiratory depression or significant sedation (22).

Numerous studies investigating the analgesic effect of dexamethasone added to local anesthetics have shown promising results (23, 24). In a study conducted by Tandoc et al., dexamethasone was found to considerably increase the duration of motor block and improve the degree of analgesia when combined with bupivacaine (25). However, dexamethasone alone did not alter the duration of analgesia or motor block (25). Preliminary studies suggest that adding dexamethasone to local anesthetics can prolong the duration of analgesia in peripheral nerve blocks (26). However, the analgesic effect of dexamethasone added to local anesthetics in TAP blocks remains debated. Consequently, the current study’s goal is to find out how well dexamethasone and dexmedetomidine work in conjunction with bupivacaine during ultrasound-guided TAP block for patients having laparoscopic cholecystectomy.

## 2. Objectives

The present study aimed to elucidate the comparative efficacy of dexmedetomidine and dexamethasone as adjuvants to bupivacaine in ultrasound-guided TAP blocks for improving postoperative pain management in patients undergoing laparoscopic cholecystectomy. Specifically, the investigation sought to determine whether the addition of these agents prolongs sensory block duration, reduces early and late postoperative pain intensity (measured via Visual Analogue Scale [VAS]), decreases 24-hour opioid consumption, and mitigates the need for rescue analgesia. Secondary objectives included evaluating the safety profiles of both adjuvants by assessing hemodynamic stability, sedation levels [using the Observer’s Assessment of Alertness/Sedation (OAA/S) Scale], and incidence of adverse events such as hypotension, bradycardia, nausea, and shivering. Furthermore, the study aimed to explore potential mechanisms underlying the observed analgesic enhancements, including the roles of α2-adrenergic receptor agonism (dexmedetomidine) and anti-inflammatory modulation (dexamethasone), while comparing their clinical utility in optimizing recovery outcomes and shortening hospital stays. By addressing these objectives, the study aimed to establish evidence-based recommendations for adjuvant selection in regional anesthesia protocols to improve postoperative recovery following minimally invasive abdominal surgeries.

## 3. Methods

The present study (registered code: IRCT20161126031095N4) was conducted as a randomized, double-blind clinical trial at Imam Khomeini Hospital in Sari, Iran, involving patients scheduled for laparoscopic cholecystectomy. Inclusion criteria comprised individuals aged 18 - 65 years with ASA class I or II who provided informed consent. Exclusion criteria included neuromuscular, hematologic, or coagulation disorders; local infection; sepsis; allergies to study medications; sleep apnea; substance abuse; uncompensated systemic diseases; psychiatric conditions; or a BMI exceeding 35.

The sample size was determined based on a power calculation to detect a 20% reduction in 24-hour morphine consumption with an α error of 0.05 and a β error of 0.2 (power = 80%), assuming a standard deviation derived from previous literature. A total of 120 ASA I/II patients were enrolled. Randomization was performed using a computer-generated random number table. Group allocation was concealed in sealed opaque envelopes. Patients, anesthesiologists administering anesthesia and performing the block, outcome assessors, and data analysts were blinded to group assignment. No interim analyses or stopping rules were planned for this trial.

Preoperatively, patients were educated on using the VAS for assessment of pain. In the operating room, an 18G intravenous catheter was inserted, and premedication with 0.03 mg/kg midazolam was administered. General anesthesia was induced using 2 µg/kg fentanyl, 2 mg/kg propofol, and 0.5 mg/kg atracurium, followed by tracheal intubation with a 7.5 mm tube for women and 8 mm tube for men, confirmed by capnography. Pressure-controlled mechanical ventilation was maintained with isoflurane-oxygen-air.

Patients were randomized into three groups (n = 40 each). Patients in the control group received 17 mL of 0.25% bupivacaine mixed with 3 mL of 0.9% saline (20 mL per side). In the Dexamethasone group, they received 17 mL of 0.25% bupivacaine combined with 4 mg dexamethasone diluted in 3 mL saline (20 mL per side). Patients in the dexmedetomidine group received 17 mL of 0.25% bupivacaine combined with 1 µg/kg dexmedetomidine diluted in 3 mL saline (20 mL per side).

Postoperatively, under aseptic conditions, a bilateral subcostal TAP block was performed in real-time using ultrasound guidance (5 - 10 MHz) by an anesthesia resident under senior supervision. The needle was inserted into the fascial plane between the internal oblique and transversus abdominis muscles, and after negative aspiration, the study drug was injected. Proper distribution was confirmed by visualizing a hypoechoic layer on ultrasound.

Patients were transferred to the post-anesthesia care unit (PACU), where patient-controlled analgesia (PCA) with morphine (loading dose: 1 mg, lockout interval: 10 minutes, maximum dose: 0.25 mg/kg over 4 hours, no basal infusion) was administered for 24 hours. Data collection included time to first opioid request, resting VAS pain scores (0 - 10), number of PCA boluses at 0, 2, 4, 6, 12, and 24 hours, total 24-hour morphine consumption (mg), nausea/vomiting severity (0 = none; 3 = vomiting), sedation levels (OAA/S Scale: 1 = awake, 5 = unresponsive), and vital signs [mean arterial pressure (MAP), oxygenation (SpO_2_), heart rate (HR), RR] recorded in PACU and at 2, 4, 6, 12, and 24 hours postoperatively. A blinded observer recorded all data.

Statistical analysis utilized Student’s *t*-test or Mann-Whitney U test for two-group comparisons, ANOVA or Kruskal-Wallis test for three-group comparisons, chi-square/Fisher’s exact test for categorical variables, and repeated-measures ANOVA for dependent variables. Analyses were performed using SPSS v26, with statistical significance set at P < 0.05.

## 4. Results

In this study, 120 patients meeting the inclusion and exclusion criteria were enrolled. The age range of participants was 30 - 65 years, with a median age of 47.5 years. The median ages in the control, dexamethasone, and dexmedetomidine groups were 48.5, 48.5, and 46 years, respectively. No statistically significant difference in age was observed between the study groups (P = 0.873). Overall, 50.8% of patients were classified as ASA class I and 49.2% as ASA class II. In the control, dexamethasone, and dexmedetomidine groups, 55.0%, 52.5%, and 45.0% of patients, respectively, were categorized as ASA class I. Similarly, there was no statistically significant difference in ASA classification distribution between the groups (P = 0.648) (Table 1). 

**Table 1.** Demographic Information and ASA Classification of Patients in Two Groups

Variables	Control Group	Dexamethasone Group	Dexmedetomidine Group	P-Value
**Median age (y)**	48.5	48.5	46	0.873
**Male (%)**	67.5	70.0	72.5	0.888
**ASA classification (Class I, %)**	55.0	52.5	45.0	0.648
**Median BMI (kg/m** ^**2**^ **)**	29.5	29.9	29.9	0.842

The median sensory block onset time in the control, dexamethasone, and dexmedetomidine groups was 5.5, 4.5, and 4 minutes, respectively. This difference was statistically significant (P < 0.001). Similarly, the median sensory block duration in the control, dexamethasone, and dexmedetomidine groups was 155, 180, and 330 minutes, respectively, with a statistically significant difference (P < 0.001). Post-hoc analysis using the Mann-Whitney U test revealed significantly longer sensory block duration in the dexamethasone group compared to the control group (P < 0.001), in the dexmedetomidine group compared to the control group (P < 0.001), and in the dexmedetomidine group compared to the dexamethasone group (P < 0.001) (Table 2). 

**Table 2.** Median Duration of Surgery, Onset and Duration of Sensory Block, Morphine Dose, and Median Length of Hospitalization

Variables	Control Group	Dexamethasone Group	Dexmedetomidine Group	P-Value
**Median surgery duration (min)**	75	75	75	0.889
**Median sensory block onset (min)**	5.5	4.5	4.0	< 0.001 ^a^
**Median sensory block duration (min)**	155	180	330	< 0.001 ^a^
**Median morphine consumption (mg)**	6	5	3	< 0.001 ^a^
**Median time to first analgesic request (min)**	350	445	530	< 0.001^ a^
**Median time to first pain report (min)**	255	340	430	< 0.001 ^a^
**Median hospital stay (d)**	2.5	2.0	1.0	< 0.001 ^a^

^a^ A P-value of less than 0.05 is considered statistically significant.

In the control, dexamethasone, and dexmedetomidine groups, 5%, 7.5%, and 25% of patients experienced hypotension, respectively. This difference was statistically significant (P = 0.013). With no statistically significant difference between the groups (P = 0.870), shivering was recorded in 5%, 7.5%, and 2.5% of patients in the control, dexamethasone, and dexmedetomidine groups, respectively. The quality of the sensory block was categorized as excellent in 20%, 32.5%, and 55% of patients; good in 35%, 30%, and 25%; and poor in 45%, 37.5%, and 20% of patients in the control, dexamethasone, and dexmedetomidine groups, respectively. There was a statistically significant difference (P = 0.021) (Table 3). 

**Table 3.** Percentage of Patients’ Symptoms in Each Group and Block Quality

Variables (%)	Control Group	Dexamethasone Group	Dexmedetomidine Group	P-Value
**Nause**	10.0	17.5	15.0	0.619
**Vomiting**	10.0	12.5	17.5	0.604
**Bradycardia**	7.5	12.5	10.0	0.928
**Hypotension**	5.0	7.5	25.0	0.013 ^a^
**Shivering**	5.0	7.5	2.5	0.870
**Pruritus**	5.0	2.5	0.0	0.772
**Excellent block quality**	20	32.5	55	0.021 ^a^

^a^ A P-value of less than 0.05 is considered statistically significant.

Assessment of pain intensity using the VAS revealed that upon admission to the PACU, pain intensity in the dexmedetomidine group was significantly lower than in the control and dexamethasone groups (P < 0.002). Subsequently, pain levels in the dexmedetomidine group remained significantly lower than the other two groups at all evaluated time points (2, 4, 6, 12, and 24 hours postoperatively) (P < 0.001). Additionally, the dexamethasone group exhibited a statistically significant reduction in pain compared to the control group, though this reduction was less pronounced than in the dexmedetomidine group.

Evaluation of oxygenation (SpO_2_) at various time points showed no statistically significant differences between the study groups (P > 0.05). Similarly, MAP at admission and across all time points demonstrated no significant intergroup differences (P > 0.05). For HR, no baseline differences were observed; however, starting 15 minutes postoperatively, HR in the dexmedetomidine group was significantly lower than in the other two groups (P < 0.001), with this reduction persisting until 60 minutes postoperatively. No significant HR differences were noted between the dexamethasone and control groups.

Assessment of patient alertness and sedation levels using the OAA/S Scale revealed that upon PACU admission, while all patients in the control and dexamethasone groups were alert, 60% of patients in the dexmedetomidine group were alert, 25% exhibited mild sedation, and 15% demonstrated moderate sedation (P < 0.001). At 2 hours postoperatively, 60% of patients in the dexmedetomidine group remained alert, and 40% showed mild sedation, whereas all patients in the other two groups remained alert. By 4 hours postoperatively and onward, all patients in all groups were alert.

## 5. Discussion

Numerous clinical studies have demonstrated that the TAP block reduces postoperative pain scores, decreases analgesic requirements, and shortens hospital stays following abdominal surgeries such as laparoscopic cholecystectomy, colectomy, appendectomy, hysterectomy, and cesarean section (27). Adequate postoperative analgesia offers significant benefits, including reduced surgical stress, lower postoperative morbidity, and improved surgical outcomes in specific procedures (28-30). Additional advantages of regional anesthesia techniques include diminished pain intensity, reduced incidence of analgesic-related complications, and enhanced patient comfort (31).

Several studies have confirmed the efficacy of TAP blocks in alleviating pain after abdominal surgeries. For example, a study on patients undergoing abdominal hysterectomy found that TAP block with ropivacaine provided superior analgesia compared to placebo for up to 48 hours postoperatively (32). Furthermore, the addition of dexmedetomidine to local anesthetics in peripheral and neuraxial blocks is recognized as an effective method to enhance anesthetic effects and reduce analgesic needs. In one study, adding 100 µg of dexmedetomidine to bupivacaine in a supraclavicular brachial plexus block extended the duration of analgesia by 8 hours (33). Another investigation reported that dexmedetomidine added to bupivacaine in TAP blocks for abdominal hysterectomy patients significantly prolonged the time to first analgesic request (470 vs. 280 minutes) and reduced 24-hour morphine consumption (19 vs. 29 mg) (16).

Multiple mechanisms have been proposed to explain dexmedetomidine’s prolonged analgesic effects, including α2-adrenergic receptor-mediated vasoconstriction, which prolongs local anesthetic efficacy (34, 35). Some studies suggest that dexmedetomidine’s action, similar to clonidine, may involve α2 receptors independent of vasoconstriction (36). The addition of dexamethasone to local anesthetics has also been shown to enhance their duration of action. In a study, 8 mg of dexamethasone added to a lidocaine-bupivacaine mixture in supraclavicular brachial plexus blocks resulted in faster onset and prolonged analgesia without significant adverse effects (37). Other studies corroborate dexamethasone’s ability to extend analgesic duration (38, 39). This effect may stem from its anti-inflammatory properties, reducing neural inflammation and hypersensitivity (40). Additionally, steroids may directly modulate neuronal membrane excitability, potentiating analgesic effects (41, 42).

Dexmedetomidine’s analgesic effect is primarily attributed to its high selectivity for α2-adrenergic receptors, which inhibits the release of norepinephrine and suppresses nociceptive transmission in the central and peripheral nervous systems. It also causes local vasoconstriction, slowing systemic absorption of local anesthetics and prolonging their effect. In contrast, dexamethasone exerts anti-inflammatory effects by suppressing phospholipase A2 activity, reducing pro-inflammatory cytokines, and modulating C-fiber activity. This may reduce perineural inflammation and prolong analgesia duration (35, 39).

In the present study, the efficacy of dexmedetomidine and dexamethasone as adjuvants to bupivacaine in TAP blocks was compared. Results demonstrated that both agents significantly reduced postoperative pain intensity compared to the control group. However, the dexmedetomidine group experienced greater pain reduction than the dexamethasone group. Moreover, the time to first analgesic request and duration of analgesia were significantly longer in the dexmedetomidine group compared to both the dexamethasone and control groups. Total 24-hour morphine consumption was lower in the dexmedetomidine and dexamethasone groups than in the control group, with the dexmedetomidine group requiring the least. The addition of dexmedetomidine to bupivacaine in TAP blocks significantly prolongs postoperative analgesia and improves pain control compared to dexamethasone or bupivacaine alone.

This study has several limitations. First, the follow-up duration was limited to 24 hours, preventing assessment of long-term analgesic efficacy or chronic pain development. Second, we did not evaluate functional recovery parameters or quality of life outcomes. Third, intraoperative opioid administration and variability in surgical technique were not standardized, which may introduce confounding factors.

## Data Availability

The dataset presented in the study is available on request from the corresponding author during submission or after publication. The data are not publicly available due to patients’ privacy.

## Clinical Trial Registration Code:

This clinical trial was registered in the Iranian Registry of Clinical Trials (IRCT) with the registration code IRCT20161126031095N4.
